# Metabolomics shows the Australian dingo has a unique plasma profile

**DOI:** 10.1038/s41598-021-84411-6

**Published:** 2021-03-04

**Authors:** Sonu Yadav, Russell Pickford, Robert A. Zammit, J. William O. Ballard

**Affiliations:** 1 School of Biotechnology and Biomolecular Sciences, University of New South Wales, Sydney, NSW 2052 Australia; 2 Bioanalytical Mass Spectrometry Facility, University of New South Wales, Sydney, NSW 2052 Australia; 3 Vineyard Veterinary Hospital, Windsor Rd, Vineyard, Sydney, NSW 2765 Australia; 4 Department of Ecology, Environment and Evolution, La Trobe University, Bundoora, VIC 3086 Australia; 5 School of Biosciences, University of Melbourne, Royal Parade, Parkville, VIC 3052 Australia

**Keywords:** Ecology, Physiology

## Abstract

Dingoes occupy a wide range of the Australian mainland and play a crucial role as an apex predator with a generalist omnivorous feeding behaviour. Dingoes are ecologically, phenotypically and behaviourally distinct from modern breed dogs and have not undergone artificial selection since their arrival in Australia. In contrast, humans have selected breed dogs for novel and desirable traits. First, we examine whether the distinct evolutionary histories of dingoes and domestic dogs has lead to differences in plasma metabolomes. We study metabolite composition differences between dingoes (n = 15) and two domestic dog breeds (Basenji n = 9 and German Shepherd Dog (GSD) n = 10). Liquid chromatography mass spectrometry, type II and type III ANOVA with post-hoc tests and adjustments for multiple comparisons were used for data evaluation. After accounting for within group variation, 62 significant metabolite differences were detected between dingoes and domestic dogs, with the majority of differences in protein (n = 14) and lipid metabolites (n = 12), mostly lower in dingoes. Most differences were observed between dingoes and domestic dogs and fewest between the domestic dog breeds. Next, we collect a second set of data to investigate variation between pure dingoes (n = 10) and dingo-dog hybrids (n = 10) as hybridisation is common in regional Australia. We detected no significant metabolite differences between dingoes and dingo-dog hybrids after Bonferroni correction. However, power analysis showed that increasing the sample size to 15 could result in differences in uridine 5′-diphosphogalactose (UDPgal) levels related to galactose metabolism. We suggest this may be linked to an increase in *Amylase 2B* copy number in hybrids. Our study illustrates that the dingo metabolome is significantly different from domestic dog breeds and hybridisation is likely to influence carbohydrate metabolism.

## Introduction

Natural selection leads to the accumulation of traits that are optimal for fitness and health in natural conditions as compared to artificial selection where organisms are selected for novel and desirable traits by humans. The Australian dingo and domestic dogs have experienced distinctive selection pressures. Dingoes arrived in Australia between 3000 and 5000 years ago^1^, are ecologically, phenotypically and behaviourally distinct from domestic dogs^2^, and can survive in the wild without human interference^3^. The dingo maintains ecosystem balance by controlling populations of introduced mesopredators and herbivores^4,5^. They are generalist predators and are widely distributed across mainland Australia^6^. The introduction of domestic dog breeds to Australia with the first fleet in 1788 initiated extensive hybridisation between dingoes and dogs^7^. Here, we study differences in plasma metabolomes between dingoes and two domestic dog breeds. We then investigate variations in plasma metabolomes between pure dingoes and dingo-dog hybrids.

Artificial selection has led to the generation of more than 400 breeds worldwide that have a diverse range of morphological, physiological and behavioural traits^8,9^. We include the Basenji and the German Shepherd Dog (GSD) as representatives of domestic dogs. We selected these two breeds because the Basenji is an ancient dog breed while the GSD has an intermediate position in the current dog phylogeny and is not morphologically specialised^10^. Historically, Basenjis were indigenous to central Africa and were used for hunting and guarding domestic herds^11^. Like dingoes, but unlike domestic dogs, Basenjis have an annual oestrus cycle^12^. On the other hand, GSDs are derived from a common livestock dog in continental Europe and were established as a unique breed in 1899^13^. GSDs are a common medium to large sized domestic dog breed, bred for their intelligence and for guarding purposes^14^. As a result of artificial selection, specific changes have occurred in genes involved in metabolism, behaviour and development^15^. For instance, the pancreatic amylase (*Amy2B*) copy number expansion in domestic breed dogs (but see^16^) is considered to be an outcome of feeding on the human provided starch rich diet^17,18^. We predict that the distinct evolutionary history of dingoes and domestic dogs, altered carbohydrate metabolism and dietary shifts in domestic dogs, and positive selection on metabolic genes result in distinct canid metabolite profiles that can be quantified.

Hybridisation between dingoes and domestic dogs has occurred since European settlement in Australia^7^ and it has led to well-established morphological and coat colour variations^2^. Interspecific hybrids can have an altered metabolite profile in their blood and urine likely as a result of genetic rearrangements and the difference in the metabolic pathways^19–21^. Hybridisation is particularly common in canids with successful inter-species reproduction and survival of fertile hybrids^22–25^. Such events can dilute the genetic pool of native populations and are a key threat to their genetic integrity^23,26^. Hybridisation may not posit a threat on the genetic integrity of wild populations if its restricted to a narrow zone between geographically widespread species. However, in the case of endangered or rare species, hybridisation can lead to genetic swamping of one population by the other, disrupt adaptive gene complexes, and reduce fitness and reproductive opportunities^27^. Here, we investigate the effects of dingo hybridisation (detected using microsatellite marker based genetic testing) on the plasma metabolome.

Metabolomics quantifies a large variety of small molecules from diverse pathways using biological samples and offers a direct link between organisms’ phenotypes and genotypes^28^. Metabolites regulate key cellular processes such as protein activity by regulating post-translational modifications, energy source and storage, membrane stabilization as well as nutrient and cell signalling^29^. Metabolite changes are readily detectable in body fluids, and provide a more direct and meaningful biochemical interpretation as compared to other ‘omics’ techniques^30^. An untargeted metabolomics approach detects the wide range of metabolites present in the sample without a priori knowledge of the metabolome composition^29^. Rapid untargeted metabolic profiling provides insights into diet associated changes in the expression of a diverse range of small molecules as shown in humans^31^. The identified metabolites (e.g., phospholipids, amino acids and vitamins) can also be used as biomarkers to inform disease progression and efficacy of clinical treatments^32–34^. The untargeted approach has been shown useful to discriminate inter- and intra-species/breed differences in domestic dogs^19,35–37^ and a study has investigated the chemical composition in dingo scat, urine and bedding^38^. To our knowledge, no studies have explored plasma metabolite profiles in dingoes.

Blood metabolite profile between individuals and species can be shaped by genetic and by environmental factors including dietary intake, physical (body) condition and gut microflora^39–43^. For instance, in several domestic dog breeds, the difference in plasma lipidome is influenced by diet under both controlled and uncontrolled dietary experiments^36,44^. In this study, we detected significant metabolite differences between dingoes and domestic dog breeds using a non-targeted plasma metabolome technique. Dingoes majorly differed from domestic dogs in protein and lipid metabolites. Further, metabolites related with galactose metabolism differed between pure dingoes and dingo-dog hybrids, but significance was lost after Bonferroni (BF) correction. Power analysis suggested an increase in sample size may lead to a significant difference in uridine 5′-diphosphogalactose (UDPgal) levels.

## Results

### Dingo and domestic breed differences

**Figure 1:**
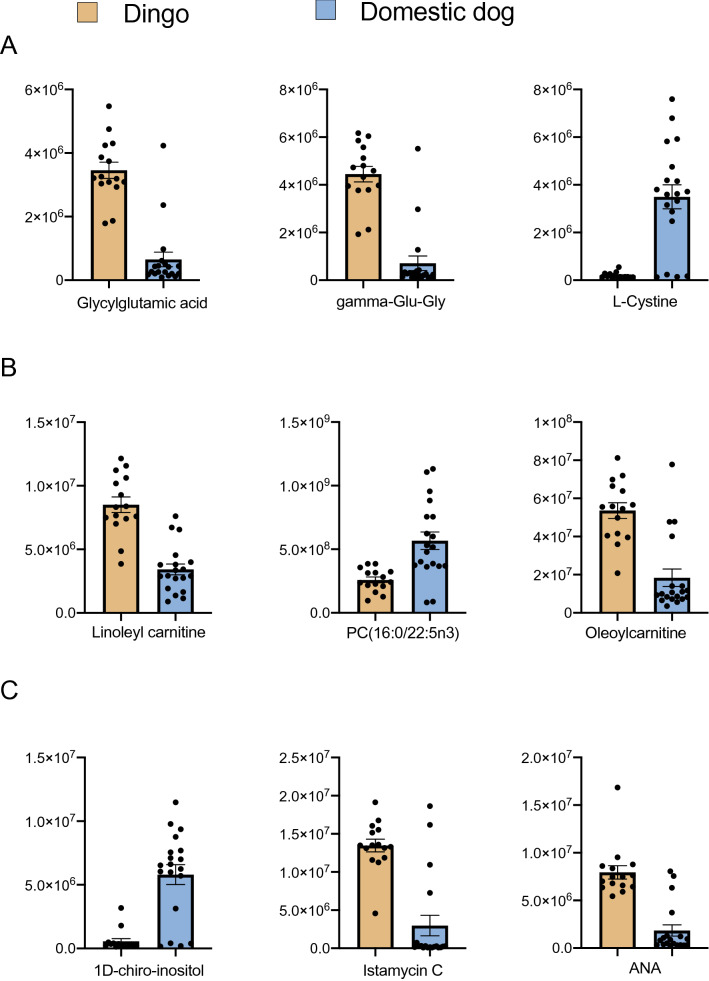
Top three metabolite differences between dingoes and domestic dog breeds jointly based on the lowest P- values: (**A**) Top three protein metabolite differences, (**B**) Top three lipid metabolite differences, (**C**) Top three carbohydrate metabolite differences. ANA: 2,7-Anhydro-alpha-N-acetylneuraminic acid (sialic acid). Y axis represents normalised area for the metabolite. Plot show mean with SE.

A total of 666 metabolites were detected by Liquid Chromatography Mass Spectrometry (LC–MS) for 34 individuals. The Type III ANOVA test identified 62 significant differences between the dingo and domestic dog (Table 1). Out of 62 metabolites, a greater number of metabolite differences were detected for protein derivatives (n = 14) followed by lipid derivatives (n = 12), carbohydrates (n = 4) (Table 1) and others (n = 32) (Table S3). Overall, the majority of proteins (71%) and lipids (66%) were lower and carbohydrates (75%) were higher in dingoes than breed dogs. For proteins, 11/14 metabolites were classified as amino acids and derivatives and 3/14 as peptides. The three protein metabolites that were most different between dingoes and domestic dogs (based on lowest P values) were Glycylglutamic acid, gamma-Glu-Gly and l-Cystine (Fig. 1A). Out of the 12 lipid differences, five were classified as phosphatidylcholines (PC) and two lysophospholipids (LyP), indicating distinction in lipid metabolism and functionality (Table 1). The three lipid metabolites with lowest P value were Linoleyl carnitine, PC (16:0/22:5n3), and Oleoylcarnitine (Fig. 1B). The three most different carbohydrate metabolites included 1D-chiro-inositol, Istamycin C and 2,7-Anhydro-alpha-N-acetylneuraminic acid (commonly known as sialic acid) (Fig. 1C).

**Table 1:** Protein, lipid and carbohydrate differences observed between the dingo and domestic dog using type III ANOVA.

Broad classification	F	P	Subclass
**Protein**			
Glycylglutamic acid	59.07	1.42E−08	Peptide
gamma-Glu-Gly	43.40	2.73E−07	Peptide
l-Cystine*	37.36	1.02E−06	Amino acid
N,N-Dimethylglycine*	36.22	1.32E−06	Amino acid derivative
D-( +)-Pipecolinic acid	34.00	2.24E−06	Amino acid metabolite
2-Amino-3-phosphonopropanoate	32.73	3.05E−06	Amino acid
Hexanoylglycine*	30.79	4.94E−06	Amino acid acylated
L-Cysteinylglycine disulphide	29.44	7.00E−06	Peptide
4-Methylene-l-glutamate*	27.72	1.10E−05	Amino acid derivative
N-Acetyl-l-leucine	27.26	1.25E−05	Amino acid derivative
2,6-Diaminoheptanedioic acid	24.85	2.43E−05	Amino acid derivative
N-acetyl-DL-tryptophan	23.70	3.38E−05	Amino acid derivative
N5-Ethyl-l-glutamine*	21.89	5.76E−05	Amino acid
Ophthalmic acid*	21.07	7.38E−05	Amino acid derivative
**Lipid**			
Linoleyl carnitine	46.29	1.51E−07	Carnitine derivative
PC (16:0/22:5n3)	32.85	2.96E−06	Phosphatidylcholine
MFCD22416941/Oleoylcarnitine	30.68	5.09E−06	Acylcarnitine
PC (32:2)	26.62	1.49E−05	Phosphatidylcholine
(2E)-hexadecenoylcarnitine	26.16	1.69E−05	Acylcarnitine
PC (18:3/18:3)	25.56	1.99E−05	Phosphatidylcholine
(24R_24′R)-Fucosterol epoxide	24.22	2.91E−05	Epoxy steroid
Nervonic acid	23.62	3.45E−05	Fatty acid
LPC (22:5)	23.18	3.93E−05	Lysophospholipid
LPC 22:6	22.73	4.48E−05	Lysophospholipid
PC (14:0/24:1)	22.62	4.64E−05	Phosphatidylcholine
PC (18:0/22:5)	21.79	5.94E−05	Phosphatidylcholine
**Carbohydrate**			
1D-chiro-inositol	31.28	4.37E−06	Sugar
Istamycin C	26.30	1.62E−05	Amino sugar
2,7-Anhydro-alpha-*N*-acetylneuraminic acid	22.97	4.19E−05	Sugar
N-Acetylneuraminic acid	22.04	5.51E−05	Amino sugar

*Non-essential amino acid derivatives and metabolites. DF = 1,30.

**Figure 2:**
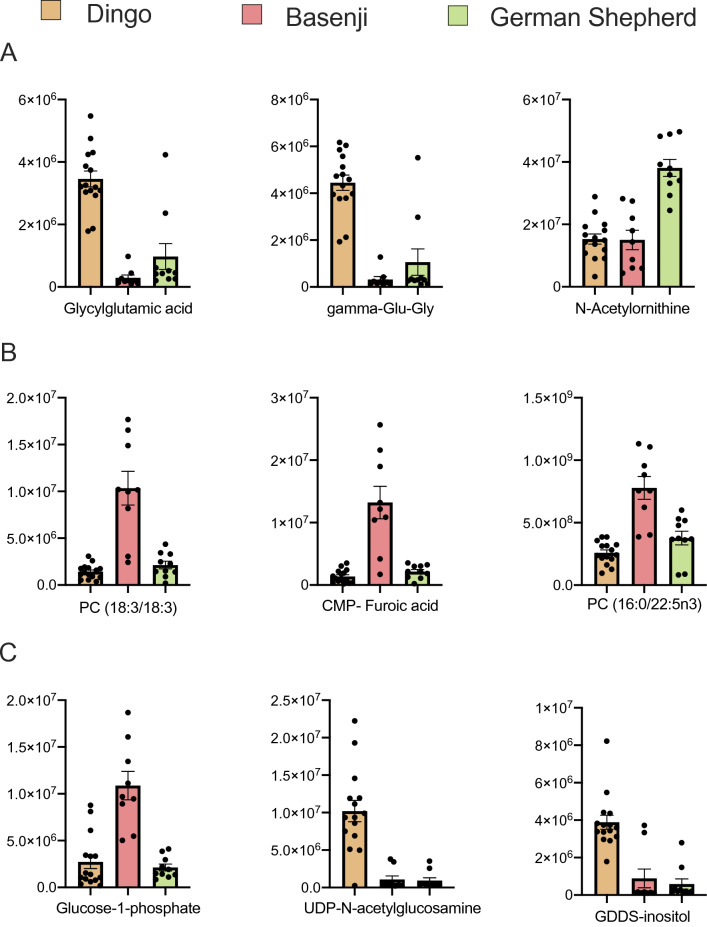
Top three metabolite differences between the dingo, Basenji and German Shepherd Dog based on the lowest P- values: (**A**) Top three protein metabolite differences between the three groups, (**B**) Top three lipid metabolite differences, (**C**) Top three carbohydrate metabolite differences. CMP- Furoic acid: 2-(2-Carboxyethyl)-4-methyl-5-pentyl-3-furoic acid, GDDS-inositol: 1D-1-guanidino-1-deoxy-3-dehydro-scyllo-inositol. Y axis represents normalised area for the metabolite. Plot show mean with SE.

Overall, ANOVA showed that 98 metabolites were significantly different between the dingo, Basenji and GSD (Table S4). A greater number of metabolite differences were detected for protein derivatives (n = 28) followed by lipid derivatives (n = 14), carbohydrates (n = 9) and others (n = 47) (Table S4). The three most different protein metabolites were Glycylglutamic acid, gamma-Glu-Gly and N-Acetylornithine (Fig. 2A). The three lipid metabolites with the greatest difference in levels were PC (18:3/18:3), 2-(2-Carboxyethyl)-4-methyl-5-pentyl-3-furoic acid, and PC (16:0/22:5n3) (Fig. 2B). The top three carbohydrate differences included Glucose-1-phosphate, UDP N-acetylglucosamine and 1D-1-guanidino-1-deoxy-3-dehydro-scyllo-inositol (Fig. 2C). Of interest, UDPgal, a metabolite associated with galactose metabolism, was significantly higher between the dingo and Basenji and between the dingo and GSD but did not differ significantly between the domestic dog breeds.

**Figure 3:**
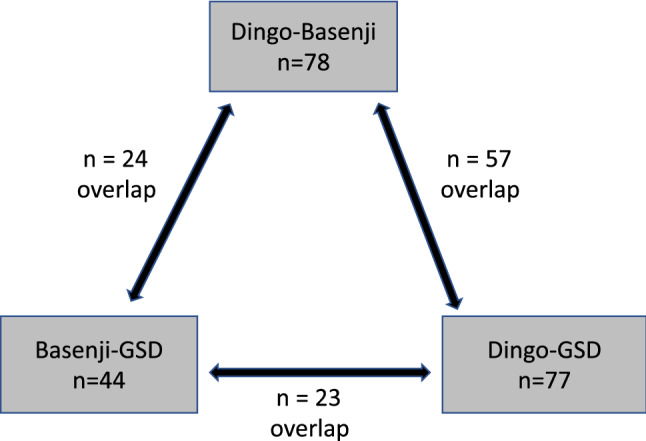
An overview of metabolite differences between the dingo, Basenji and German Shepherd Dog (GSD) detected using pairwise Tukey’s test. Each grey box represents metabolite differences for the respective pair and overlap represents the number of common metabolites detected between each group.

Tukey’s test showed significant pairwise metabolite differences between dingoes and Basenjis (n = 78), dingoes and GSDs (n = 77), with fewer significant metabolite differences between Basenjis and GSDs (n = 44) (Fig. 3). Between dingoes and Basenjis there were 21 unique metabolites that differed (Table S5), 20 between dingoes and GSDs (Table S6), and no unique metabolites between Basenjis and GSDs. Comparing the dingo and Basenji, 10 lipid metabolites differed and all were lower in dingoes. In contrast, the dingo and GSD differed in 11 protein metabolites, again all lower in the dingo.

### Pure and hybrid dingo differences

**Figure 4:**
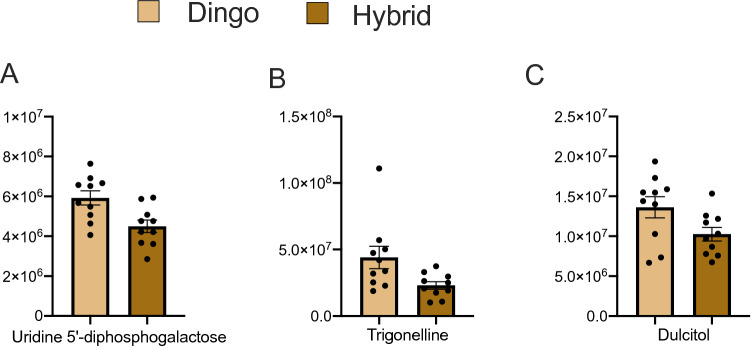
Metabolite difference between the dingo and dingo-domestic dog hybrid. Y axis represents normalised area for the metabolite. Plot show mean with SE.

A second LC–MS analysis on 10 dingoes and 10 dingo-dog hybrids was conducted and a total of 143 metabolites were detected. Out of these, UDPgal (t_(17.7)_ = − 3.01, P uncorrected = 0.0075), trigonelline (t_(11.03)_ = − 2.37, P uncorrected = 0.037), dulcitol (t_(15.3)_ = − 2.13, P uncorrected = 0.049), taurine (t_(17.52)_ = − 3.73, P uncorrected = 0.002), and l-Glutathione oxidized (t_(14.46)_ = − 2.33, P uncorrected = 0.03) had significantly higher levels in pure dingoes. BF correction, however, resulted in loss of significance in all cases. A post-hoc power test indicated a sample size of 15, 24 and 29 individuals respectively would result in a significant difference for UDPgal, trigonelline, and dulcitol (Fig. 4).

## Discussion

Dingoes are Australia’s apex predator and their natural history is extensively studied^3^. However, little is known about their cell biology or metabolic profile^38^. Our study reveals significant differences in the plasma metabolite composition between the dingo and domestic dogs. Of the 62 significant differences between the dingo and domestic dogs 71% of protein metabolites and 66% of lipid metabolites were lower in dingoes. The relatively low protein and lipid metabolite levels in dingoes may reflect genetic, body condition or dietary differences. We support the former explanation as we included dingoes and breed dogs from multiple sources. Comparing dingoes and dingo-dog hybrids, where animals were maintained in the same environmental conditions, metabolites associated with galactose metabolism were significantly higher in pure dingoes before BF correction. Our results provide insight into how the dingo and the domestic dog, with their distinct evolutionary histories, show variations in the cellular and metabolic pathways.

Metabolic differences involved in crucial pathways such as immune functioning and neurodevelopment indicate that the ~ 8000 years of divergence of the dingo from domestic dogs have affected key genes and their metabolites essential for survival and fitness. Dingoes are generalist predators and a large proportion of the dingo diet includes protein^6^. A high protein diet may reinforce metabolites related to protein digestibility in the dingo compared to the domestic dog, which consumes food with high starch and low animal protein^45^. In our study comparing dingoes with domestic dogs, six protein derivatives that differed between dingo and domestic dogs are derived from non-essential amino acids, which are produced internally (Table 1). These protein derivative differences support our hypothesis that there are underlying genetic differences between dingoes and dogs. A study on the dingo reported that 50 candidate genes associated with digestion and metabolism are under positive selection^46^.

We identified multiple metabolites that are associated with neurodevelopment and likely linked with the process of domestication. The glutamate receptor agonist 2-Amino-3-phosphonopropanoate was lower in dingoes. Critically, this agonist has been shown to influence neurotransmission^47^. The unsaturated fatty acid nervonic acid was also lower in dingoes than domestic dogs. Nervonic acid is tightly linked with brain development, improving memory, delaying brain aging and biosynthesis of nerve cells^48^. The carbohydrate sialic acid was higher in dingoes and is essential for mediating ganglioside distribution and structures in the brain^49^. Previously, Wang, et al.^50^ showed that six genes associated with the glutathione metabolism and 49 genes associated with the neurological process and perception are under positive selection during dog domestication.

In our study, we observed significantly different levels of three protein metabolites that are associated with the bacterial community in the gastrointestinal tract. Dingoes had lower levels of protein N-acetyl-DL-tryptophan, and 2,6-Diaminoheptanedioic acid and higher levels of D-pipecolic acid. N-acetyl-DL-tryptophan is a tryptophan catabolite converted by gut microbiota^51^. It is also a protein stabilizer and protects protein molecules from oxidative degradation. 2,6-Diaminoheptanedioic acid is a lysine like derivative and is a key component of the bacterial cell wall^52^. It can be found in the body fluids as a result of the enzymatic breakdown of gram-negative gut microbes. D-pipecolic acid is produced from the metabolism of intestinal bacteria^53,54^. We predict dingoes and domestic dogs will differ in their gut microbiome composition and suggest future gut microbial studies of dingoes and domestic dogs should explore animals in their natural habitats as well as fed on controlled diets as nutrition can influence the gut microbiota.

Additional metabolite differences between the dingo and domestic dog detected a suite of metabolites that influence cell signalling and immune system functioning. Of interest, the dipeptide gamma- Glu-Gly was elevated in dingoes. Glu-Gly is an excitatory amino acid receptor antagonist in the hippocampus^55^. l-Cystine, lower in dingoes, is an oxidised form of cysteine and is linked with the immune system. l-Cystine is the preferred form of cysteine for the synthesis of glutathione in immune system cells such as macrophages and astrocytes. The vitamin DL-alpha-tocopherol was lower in dingoes. It is important for regulating immune function^56^. We thus propose that immune responses are expected to be higher in the dingo than the domestic dog because they are exposed to a range of environments and there is relaxed selection for high immunity in domestic dogs due to increased Veterinary intervention.

Among lipids, both LyP and all five PCs were lower in dingoes than domestic dogs (Table 1). LyP are important for cell membrane biosynthesis, energy source and storage and intracellular signalling by acting on LPL-R lysophospholipid receptors^57^. In addition, LyPs are involved in several fundamental processes such as reproduction, nervous system function and immunity^58,59^. PCs are the predominant component of mammalian cell membranes^60^ and are involved in the regulation of lipid, lipoproteins, and energy metabolism^61,62^. Combined, the data presented in this study indicates that pure dingoes have a distinct organismal functions and physiology compared to feral domestic dogs that result in increased immune system functioning and neurotransmission.

The second study compared pure dingoes with hybrids. The data obtained suggests significant differences may be detected for UDPgal, dulcitol, and trigonelline after increasing the sample size. Consistent with this interpretation, our concurrent analysis also detected a significant difference in UDPgal levels between dingoes and breed dogs (Table S4). UDPgal and dulcitol are produced from galactose metabolism^63^. Both metabolites were higher in pure dingoes than hybrids (Fig. 4), putatively a result of lower metabolic digestion of galactose in dingoes. Potentially, this could be linked with the low *Amy2B* copy number in pure dingoes^18^. Like wolves, most of not all, pure dingoes have a single copy of *Amy2B*. In contrast, domestic dogs have an expansion of *Amy2B* that is linked with increased carbohydrate metabolism and likely effects feeding behavior^64,65^. Domestic dogs are attracted to several sugars including sucrose, glucose, lactose and fructose, and have a high carbohydrate metabolic potential^66,67^. Admixture between genes from domestic dog breeds in the dingo can form new genetic combinations influencing the expression of genes involved in the carbohydrate metabolism, which likely influences their behaviors.

Future studies including East Asian breed dogs and additional hybrids will test the hypotheses presented here. Most recently, dingoes have been shown to form a monophyletic clade with East Asian breed dogs^68^. We do not know the history of the hybrid dingoes included in this study. Including dingo-dogs hybrids with different levels of distinct domestic breeds is needed to determine whether the differences in galactose metabolism are due to increases on *Amy2B* copy number or changes in allele frequency of other genes. Future studies should quantify *Amy2B* copy number. Further, running authentic standards (i.e. positive controls) confirming the identity of key metabolites would strengthen our confidence in the metabolite characterization. While significant differences in the plasma metabolome were detected using robust univariate statistical analyses, the multivariate exploration of the data and a greater sampling effort in future studies would further strengthen our conclusions.

## Conclusion

Our findings demonstrate that plasma metabolite profiling can be used to capture metabolome differences between the dingo and domestic dog breeds despite diet and environmental variability. Our results are consistent with the expectation that the distinct evolutionary history of dingoes and domestic dogs has played an important role in shaping pathways linked with protein, lipid and carbohydrate metabolism. A vast number of detected metabolite differences between dingoes and domestic dogs were involved in immune system functioning and neurotransmission indicating differential selection pressure on pathways crucial for fitness and survival. By comparing the pure and hybrid dingoes reared under similar environmental conditions and food, we showed that hybridisation might lead to differences in metabolites involved in the carbohydrate metabolic pathways.

## Materials and methods

### Sampling and included animals

To test for differences between dingoes and the domestic breeds 34 individuals were included. Ten dingoes were collected from Bargo dingo sanctuary in south-eastern Australia. Five additional dingoes from diverse geographic localities throughout Australia were included to test the generality of the results. Dingoes from sanctuaries were either born in the wild but humanized before six weeks of age or were sanctuary born. All dingoes had daily interactions with humans, were fed daily and kept in a lean condition. For the domestic dogs, we included nine Basenjis from two kennels, and 10 GSDs from two kennels. All domesticates were kept in a lean condition and none were overweight. All kennels were in south-eastern Australia (Table S1). The animals were between 1–10 years and closely matched for sex but unmatched on diet to keep consistency with natural conditions.

To test for differences between pure dingoes and dingo-dog hybrids a second set of 10 pure and 10 hybrid dingoes collected from the same locality (Table S2). All 20 canines were aged from 1 to 12 years and maintained under same environmental conditions. The individuals were diet and sex matched with equal numbers of males and females. Additional samples could not be included without extreme bias of the sample design (age, purity and sex).

The purity of all dingoes and hybrid dingo status was established using the 23 microsatellite marker based dingo purity genetic test^69^. Basenjis and GSDs were purebred and registered with the Australian Kennel Club.

### Metabolite extraction

Blood samples were immediately stored in EDTA tubes to avoid clotting. Plasma was separated from frozen and fresh whole blood by centrifuging at 2,000 g for 10 min at 4 °C. Immediately after centrifugation, plasma was transferred into clean microtubes and stored at − 80 °C for further processing.

Samples were extracted following Mackay, et al.^70^. Briefly, 10 µL of thawed plasma samples were diluted 20-fold with cold extraction solvent (50% methanol, 30% acetonitrile, 20% water at approximately − 20 °C). To mix and remove any proteins, samples were vortexed for 30 s, and then centrifuged at 23,000 g for 10 min at 4 °C. The supernatants were transferred to glass HPLC vials and kept at − 80 °C prior to LC–MS analysis. Pooled quality control samples were created by combining 5 µL of each sample. Process blanks were created by following the extraction protocol without plasma.

Liquid chromatography-mass spectrometry (LC–MS) profiling was performed using Q-Exactive HF Mass Spectrometer with U3000 UHPLC system (ThermoFisher Scientific). Samples were analysed in both positive and negative heated electrospray ionization as separate injections. Samples and blanks were analysed in a random order (generated using Excel) with regular QC’s inserted into the sequence after randomisation. Samples were run in two batches, the first batch included 34 individuals (15 dingoes, 9 Basenjis and 10 GSDs) and the second batch included 20 individuals (10 pure and 10 hybrid dingoes). All statistical tests were performed within the same batch.

A ZIC-pHILIC column (SeQuant, VWR, Lutterworth, Leics., UK) was employed to measure a broad range of metabolites of different classes as it is suggested to give the broadest coverage of metabolites with an adequate performance as compared to the other columns^71^. 5 µL of the sample was injected onto the column. Separation was performed using a gradient of mobile phase A (20 mM ammonium carbonate in MilliQ water, adjusted to pH 9.4 with ammonium hydroxide) and mobile phase B (100% acetonitrile) at 200 µL/min. The gradient was held at 80% B for 2 min, ramped to 20% B at 17 min before returning to 80% B at 17.1 min and holding for re-equilibration until 25 min. The mass spectrometer was operated in the data dependant analysis mode—automatically acquiring MS/MS data. The instrument was scanned from 75 to 1000 at a resolution of 60 K, with MS/MS of the top 20 ions at 15 K. Source conditions were spray voltage 4.5 kV positive, (3.5 kV negative), sheath gas 20au, auxiliary gas 5au. Heater temperature was 50 °C and the capillary temperature was 275 °C. S-Lens was 50 V. The instrument was calibrated immediately prior to data acquisition and lock masses used to maintain optimal mass accuracy.

Data analysis was performed using Compound Discoverer software (v3.1 Thermo, Waltham, USA). The software was used to pick and integrate peaks, perform relative quantitation and attempt identification using database searches against mzCloud and Chemspider databases. The QC samples were used to correct chromatographic drift and the processed blanks used to identify and filter out background components. Before statistical analysis the data was filtered and only the most confident identifications (> 50% score against mzCloud) were used. Normalised area, to compensate for the change in mass spectrometer signal across time, for each metabolite was exported to excel format and then used for further statistical analysis. Metabolite classification and functions were determined using Human Metabolome Database (HMDB) and PubChem database.

### Statistical analyses

All statistical analyses were performed in R v3.6.1^72^. An overall significant difference in the metabolites between dingoes and domestic dogs (Basenji and GSD) was determined by performing Type III ANOVA to account for within group variation. To detect differences between the dingo, Basenji and GSD a Type II ANOVA was performed using car R package^73^. Following ANOVA we obtained the pairwise difference between groups using *TukeyHSD* function in R. To identify metabolite difference between pure and hybrid dingoes a Welch two sample t-test was performed. A post-hoc power test was then performed using the *pwr.t.test* function in R^74^ with a significance at P = 0.05 and power of 95%. All P- values obtained from statistical tests were BF corrected to account for multiple comparisons. All statistical analyses were performed on the combined positive ion and negative ion data sets.

### Ethical approval

All procedures were conducted in accordance with the guidelines of University of New South Wales and were approved by University of New South Wales Ethics Approval ID’s 16/77B, 18/148B and ACEC ID: 18/18B to J.W.O.B. The dingo blood samples were sent to University of New South Wales for dingo genetic purity testing under the ethic permit. Informed consent was obtained from the owner of the animal wherever required.

## Supplementary Information

Supplementary Information 1.

Supplementary Information 2.

## Data Availability

Data files are in supplementary materials.
