# Supplementary material for: Metabolomics shows the Australian dingo has a unique plasma profile
Source: Sci Rep. 2021 Mar 4;11:5245. doi: 10.1038/s41598-021-84411-6 (PMC7933249; doi:10.1038/s41598-021-84411-6)
Supplement: Supplementary file 1 — Supplementary Information 1. [file 41598_2021_84411_MOESM1_ESM.pdf]

Supplementary information for

Metabolomics shows the Australian dingo has a unique plasma profile

Sonu Yadav[1]*, Russell Pickford[2], Robert A. Zammit[3], J. William O. Ballard[4,5]

[1]School of Biotechnology and Biomolecular Science, University of New South Wales, Sydney, NSW, 2052, Australia

[2]Bioanalytical Mass Spectrometry Facility, University of New South Wales, Sydney, NSW, 2052, Australia

[3]Vineyard Veterinary Hospital, Windsor Rd, Vineyard, Sydney, NSW 2765, Australia

[4] Department of Ecology, Environment and Evolution, La Trobe University, Bundoora, Victoria 3086, Australia

[5] School of Biosciences, University of Melbourne, Royal Parade, Parkville, Victoria 3052, Australia

***Corresponding author:**

Sonu Yadav

School of Biotechnology and Biomolecular Sciences, University of New South Wales, Sydney, NSW 2052, Australia. E-mail: Sonu.yadav@unsw.edu.au

Table S1: Details of canines included to detect metabolite differences between dingoes and domestic breeds. NSW= New South Wales, WA= Western Australia, QLD = Queensland.

Local ID	Group	Sex	Age	Location
W0381	Dingo	F	4	Bargo dingo sanctuary, NSW
W0380	Dingo	M	4	Bargo dingo sanctuary, NSW
W0378	Dingo	F	3	Bargo dingo sanctuary, NSW
W0379	Dingo	F	3	Bargo dingo sanctuary, NSW
X3170	Dingo	M	8	Bargo dingo sanctuary, NSW
W0235	Dingo	M	4	Bargo dingo sanctuary, NSW
W0302	Dingo	M	5	Bargo dingo sanctuary, NSW
W0363	Dingo	M	1	Bargo dingo sanctuary, NSW
W0383	Dingo	F	3	Bargo dingo sanctuary, NSW
X3172	Dingo	M	3	Bargo dingo sanctuary, NSW
W0296	Dingo	F	4	Pure dingo sanctuary, NSW
W0330	Dingo	F	4	Pure dingo sanctuary, NSW
W0349	Dingo	F	1	Crossroads Dingo Rescue, WA
W0351	Dingo	F	>1	RSPCA, Victoria
W0358	Dingo	M	2	Mandurah, WA
BAS06	Basenji	F	2.5	Basenji breed network, QLD
BAS07	Basenji	M	6.8	Basenji breed network, QLD
BAS22	Basenji	M	6.7	Zanzipow Basenji club, NSW
BAS23	Basenji	F	4	Zanzipow Basenji club, NSW
BAS24	Basenji	F	4	Zanzipow Basenji club, NSW
BAS25	Basenji	F	10	Zanzipow Basenji club, NSW
BAS26	Basenji	M	5.7	Zanzipow Basenji club, NSW
BAS27	Basenji	F	10	Zanzipow Basenji club, NSW
BAS28	Basenji	M	7	Zanzipow Basenji club, NSW
BAS29	Basenji	F	2.6	Zanzipow Basenji club, NSW
GSD03	German Shepherd	M	2	Allendelle Kennel, NSW
GSD06	German Shepherd	F	1.8	Kingsvale Kennel, NSW
GSD07	German Shepherd	F	1.8	Kingsvale Kennel, NSW
GSD08	German Shepherd	F	3.6	Kingsvale Kennel, NSW
GSD 11	German Shepherd	M	2.2	Kingsvale Kennel, NSW
GSD12	German Shepherd	F	2.1	Kingsvale Kennel, NSW
GSD14	German Shepherd	M	5.6	Kingsvale Kennel, NSW
GSD15	German Shepherd	F	4.1	Kingsvale Kennel, NSW
GSD16	German Shepherd	M	4	Kingsvale Kennel, NSW
GSD 17	German Shepherd	F	>1	Kingsvale Kennel, NSW

Note: No sedatives were utilised on any dingo during blood collection. Dingoes were gently restrained by a known handler and blood was extracted quickly to avoid even the slightest rise in blood cortisol. Blood was drawn from the cephalic vein.

Table S2: Details of dingo-dog hybrids and pure dingoes. NSW= New South Wales

Local ID	Group	Sex	Age	Location
W0439	Hybrid dingo	F	1	Western NSW
W0431	Hybrid dingo	M	6	Western NSW
W0446	Hybrid dingo	F	4	Western NSW
W0442	Hybrid dingo	F	3	Western NSW
W0456	Hybrid dingo	M	2	Western NSW
W0445	Hybrid dingo	F	1	Western NSW
W0458	Hybrid dingo	M	2	Western NSW
W0429	Hybrid dingo	M	8	Western NSW
W0427	Hybrid dingo	M	8	Western NSW
W0457	Hybrid dingo	F	1	Western NSW
W0435	Pure dingo	M	12	Western NSW
W0437	Pure dingo	F	4	Western NSW
W0449	Pure dingo	M	10	Western NSW
W0454	Pure dingo	M	3	Western NSW
W0450	Pure dingo	M	8	Western NSW
W0440	Pure dingo	F	1	Western NSW
W0448	Pure dingo	F	1	Western NSW
W0453	Pure dingo	F	3	Western NSW
W0433	Pure dingo	M	1	Western NSW
W0452	Pure dingo	F	1	Western NSW

Table S3: Metabolite differences between the dingo and domestic dog detected using Type III ANOVA analysis. Basenji and German Shepherd Dog were grouped together as domestic dogs. DF= 1,30.

Broad classification			
**Other**	F	P	Subclass
Pseudouridine	23.73	3.35E-05	Nucleoside and nucleotide analogues
Orotidine	21.79	5.93E-05	Pyrimidine nucleoside
3',5'-Cyclic IMP	34.14	2.17E-06	Nucleotide
Cangrelor	28.20	9.71E-06	Nucleoside triphosphate analogue
3'-Adenosine monophosphate (3'-AMP)	22.16	5.31E-05	Nucleotide
Arabinosylhypoxanthine	36.85	1.14E-06	Purine nucleoside
2-Aminonicotinic acid - Vitamin	46.34	1.50E-07	Vitamin B3 derivative
DL-α-Tocopherol/ Vitamin E	47.08	1.29E-07	Tocopherol
Mebutamate	36.68	1.19E-06	Synthetic
PEG n10/Polyethylene glycol (PEG)	25.58	1.98E-05	Synthetic polyether
Amfepramone	71.53	1.94E-09	Syntheic, Drug
Ethylenediaminetetraacetic acid	34.76	1.87E-06	EDTA synthetic
4-Amino-5-hydroxymethyl-2-methylpyrimidine	24.57	2.64E-05	Aminopyrimidine
6-Methoxyquinoline	23.17	3.94E-05	Aromatic Ether and quinoline
Nitrilotriacetic acid	43.96	2.43E-07	Carboxylic acid derivative
N-(6-Oxo-6H-dibenzo[b,d]pyran-3-yl)maleamic acid	21.78	5.96E-05	Coumarin member
Carpropamid	28.56	8.81E-06	Cyclopropylcarboxamide
3-Hydroxy-3-[(3-methylbutanoyl)oxy]-4-(trimethylammonio)butanoate	21.73	6.05E-05	Derived from by product of leucine degradation pathway
Taxifolin	24.36	2.79E-05	Flavonoid
Benzimidazole	31.00	4.69E-06	Imidazole derivative
1D-1-Guanidino-1-deoxy-3-dehydro-scyllo-inositol	21.68	6.15E-05	Inositol derivative-sugar
Triadimefon	39.36	6.50E-07	Triazoles member
N-Nitrosoguvacoline	22.87	4.30E-05	*N*-nitrosamine
4-(3-Hydroxybutyl)-2-methoxyphenyl hydrogen sulfate	26.95	1.36E-05	Phenylsulfates
Hexahydroxydiphenic acid	26.34	1.60E-05	Polyphenol
1-{[5-(2-Hydroxyethoxy)-4-oxopentanoyl]oxy}-2,5-pyrrolidinedione	21.59	6.31E-05	Secondary amine
[FAoxo_amino(6:0)]3-oxo-5S-amino-hexanoicacid	38.85	7.28E-07	Keto acids and derivative
**Unknown**			
6-[(5-Amino-1-carboxypentyl)amino]-3,4,5-trihydroxytetrahydro-2H-pyran-2-carboxylic acid	44.67	2.10E-07	Unknown
(1R,9S)-11-[(Methylsulfanyl)acetyl]-3-(2-thienyl)-7,11-	33.27	2.67E-06	Unknown

diazatricyclo[7.3.1.02,7]trideca-2,4-dien-6-one			
5-(2,6-Dichlorobenzyl)-6-methyl-2-(2-pyridyl)pyrimidin-4-ol	23.48	3.60E-05	Unknown
5,5'-Dihydroxy-4,4',8',8'-tetramethyl-4,5-dihydro-2'H,3H-spiro[furan-2,6'-[7]oxabicyclo[3.2.1]oct[3]en]-2'-one	21.79	5.94E-05	Unknown
X7028	40.21	5.39E-07	Unnamed

Table S4: Table showing 98 metabolite differences between the dingo, Basenji and German Shepherd Dog (GSD) using type II ANOVA and pairwise differences between groups obtained from Tukey's test. DF= 2,31. P represents P-value obtained from type II ANOVA.

Metabolite	F	P	Dingo vs Basenji	Dingo vs GSD	GSD vs Basenji
**Protein**					
4-Hydroxyphenylacetylglycine	17.62	7.74E-06	8.59E-05	6.26E-05	0.998951932
L-Cystine	17.49	8.23E-06	0.000805194	1.41E-05	0.490665829
2-Amino-3-phosphonopropanoate	17.21	9.38E-06	3.44E-05	0.000252339	0.721165446
4-Methylene-L-glutamate	15.61	2.04E-05	6.81E-05	0.000477632	0.731297732
L(+)-Citrulline	14.45	3.68E-05	0.35783856	2.46E-05	0.004463141
N5-Ethyl-L-glutamine	14.24	4.11E-05	0.13272287	2.39E-05	0.017329319
Hypoglycin A	14.14	4.33E-05	7.58E-05	0.996775377	0.000290317
Hexanoylglycine	16.76	1.16E-05	0.011702382	8.16E-06	0.078400491
N-Acetylornithine	29.68	6.29E-08	0.996027099	1.81E-07	1.31E-06
2_6 Diamino heptanedioic acid	19.90	2.76E-06	0.222246263	1.73E-06	0.00102609
L-Homocitrulline	19.24	3.69E-06	0.589745147	3.33E-06	0.000296742
N,N-Dimethylglycine	18.98	4.15E-06	0.007140673	2.88E-06	0.057509905
(3S)-3_6-Diaminohexanoate	17.71	7.43E-06	0.547636654	6.22E-06	0.000610986
2-Methylserine	16.54	1.29E-05	1.09E-05	0.006095366	0.089699004
N-Acetyl-L-leucine	15.83	1.83E-05	0.042032494	1.09E-05	0.032230509
N(5)-(L-1-carboxyethyl)-L-ornithine	14.55	3.49E-05	0.608533237	2.92E-05	0.001788435
Ophthalmic acid	13.42	6.35E-05	0.000260737	0.000758569	0.885432446
D-(+)-Pipecolinic acid	17.54	8.02E-06	0.000208132	3.15E-05	0.891670479
6-Acetamido-3-aminohexanoate	15.56	2.09E-05	0.078925629	1.20E-05	0.018403719
L-Alanyl-L-proline	18.57	4.99E-06	0.891892444	6.75E-06	0.000139746
Glycylglutamic acid	35.76	8.88E-09	3.37E-08	1.90E-06	0.294404999
gamma-Glu Gly	35.52	9.55E-09	5.01E-08	1.19E-06	0.439644045
L-Glutathione (reduced)	19.24	3.69E-06	4.20E-05	3.59E-05	0.994421806

gamma-Glu gln	16.35	1.42E-05	0.622704423	9.86E-05	3.98E-05
val glu	15.67	1.98E-05	0.000477733	6.52E-05	0.870971035
L-Cysteinylglycine disulfide	15.12	2.61E-05	0.00999075	2.12E-05	0.156976629
Gly Pro Glycylproline	14.90	2.92E-05	0.266403734	1.83E-05	0.005607749
pro gln	13.73	5.37E-05	3.22E-05	0.201280392	0.00777949
**Lipid**					
Cytidine	19.37	3.48E-06	4.33E-06	0.000963264	0.164723235
(2E)-hexadecenoylcarnitine	19.49	3.30E-06	0.000363231	6.38E-06	0.499863217
MFCD22416941	15.33	2.35E-05	9.84E-05	0.000388888	0.835205992
2-(2-Carboxyethyl)-4-methyl-5-pentyl-3-furoic acid	26.61	1.87E-07	2.95E-07	0.888601613	4.63E-06
Diallyl adipate	21.82	1.21E-06	4.81E-06	0.891189447	6.01E-06
Nervonic acid	21.04	1.69E-06	1.05E-06	0.203753158	0.000389254
14(Z)-Eicosenoic acid	20.75	1.91E-06	2.95E-06	0.93026158	3.07E-05
cis-5,8,11,14,17-Eicosapentaenoic acid	18.57	4.99E-06	0.000132903	0.219642801	5.39E-06
Docosahexaenoic acid	16.98	1.05E-05	4.80E-05	0.799514938	3.13E-05
Linoleyl carnitine	24.22	4.63E-07	3.16E-06	1.42E-05	0.79647335
LysoPC (22:1(13Z))	21.13	1.63E-06	3.34E-06	0.995669677	1.72E-05
1,2-di-[(9Z,12Z,15Z)-octadecatrienoyl]-sn-glycero-3-phosphocholine	29.98	5.67E-08	8.36E-08	0.818406692	2.00E-06
1-hexadecanoyl-2-[(7Z,10Z,13Z,16Z,19Z)-docosapentaenoyl]-sn-glycero-3-phosphocholine	24.60	4.00E-07	2.73E-07	0.248090621	8.26E-05
LPC(22:6)	15.45	2.21E-05	1.30E-05	0.167938946	0.004632365
**Carbohydrate**					
N-Acetylneuraminic acid	24.60	4.00E-07	0.000119183	6.89E-07	0.317317543
Glucose-1-phosphate	25.20	3.18E-07	1.37E-06	0.878691883	1.78E-06
Istamycin C	19.64	3.09E-06	0.000204367	8.06E-06	0.659697612
Benzoyl glucuronide (Benzoic acid)	17.75	7.29E-06	0.781717133	8.05E-06	0.000284895
Aminoimidazole ribotide	14.56	3.47E-05	0.00141857	0.142093067	2.67E-05

Uridine 5'-diphosphogalactose	14.54	3.52E-05	0.000477691	0.000154309	0.970738845
1D-chiro-inositol	17.07	1.00E-05	3.09E-05	0.000337774	0.643049757
2,7-Anhydro-alpha-N-acetylneuraminic acid	15.12	2.62E-05	0.000423394	0.000108177	0.950466643
1D-1-Guanidino-1-deoxy-3-dehydro-scyllo-inositol	23.95	5.14E-07	2.14E-05	2.70E-06	0.877081894
**Other**					
beta-Nicotinamide mononucleotide	19.53	3.25E-06	7.73E-05	1.71E-05	0.942992496
Pseudouridine	13.29	6.80E-05	0.007362303	7.34E-05	0.35442431
Cangrelor	13.23	7.02E-05	0.000577774	0.000389073	0.999994364
3'-Adenosine monophosphate (3'-AMP)	25.47	2.87E-07	6.15E-05	6.13E-07	0.407389452
3',5'-Cyclic IMP	18.65	4.81E-06	1.36E-05	0.000241288	0.545320856
Uric acid	13.12	7.42E-05	4.56E-05	0.068308965	0.034132044
Arabinosylhypoxanthine	35.83	8.71E-09	1.45E-07	2.53E-07	0.920912441
Orotidine	17.49	8.21E-06	0.000480318	1.87E-05	0.648886247
N4-Acetylcytidine; N-Acetyl-Cytidine	16.12	1.58E-05	1.76E-05	0.81265826	0.000298371
UDP N-acetylglucosamine	24.11	4.84E-07	1.01E-05	4.54E-06	0.996112259
DL-α-Tocopherol - Vitamin	20.18	2.44E-06	2.60E-05	2.84E-05	0.981836759
2 Aminonicotinic acid - Vitamin	34.28	1.40E-08	7.57E-09	0.001664877	0.000644166
N-((4-AMINO-2-METHYL-5-PYRIMIDINYL)METHYL)FORMAMIDE	14.14	4.33E-05	0.369376643	2.91E-05	0.004855688
4-Amino-5-hydroxymethyl-2-methylpyrimidine	18.51	5.13E-06	0.218698958	3.17E-06	0.001762667
6-Methoxyquinoline	31.52	3.38E-08	8.06E-08	0.992551967	5.71E-07
6-Hydroxypseudooxynicotine	20.11	2.51E-06	0.657465888	2.49E-06	0.000172797
D-1,2,3,4-Tetrahydroisoquinoline-3-carboxylic acid	16.58	1.27E-05	0.617956701	1.12E-05	0.000760747
Nitrilotriacetic acid	20.14	2.48E-06	3.80E-06	0.000486335	0.224327011
Carpropamid	18.00	6.47E-06	1.08E-05	0.000726789	0.312753332
Nitrendipine	21.97	1.14E-06	1.15E-05	1.75E-05	0.953528123
Amfepramone	61.83	1.52E-11	1.00E-09	3.43E-10	0.992159831
Dimetridazole	23.75	5.56E-07	5.47E-06	0.528480577	1.39E-06

Propamocarb	20.46	2.16E-06	0.970446103	6.31E-06	2.17E-05
Ethylenediaminetetraacetic acid	18.99	4.13E-06	1.95E-05	0.000104162	0.776963989
Taxifolin	14.30	3.99E-05	5.89E-05	0.002742279	0.367511144
8-Hydroxyalanylclavam	14.10	4.42E-05	3.21E-05	0.020184768	0.076069401
5-Nonyl-2-oxotetrahydro-3-furancarboxylic acid	15.82	1.84E-05	1.15E-05	0.248428052	0.002435442
Benzimidazole	20.91	1.79E-06	7.64E-05	6.98E-06	0.820353547
Methylimidazoleacetic.acid	27.73	1.25E-07	0.792591801	6.90E-07	1.01E-06
[FAoxo_amino(6:0)]3-oxo-5S-amino-hexanoicacid	27.86	1.19E-07	0.003567317	6.76E-08	0.005863915
2-Amino-5-[2-(4-formylphenyl)hydrazino]-5-oxopentanoic acid	13.55	5.91E-05	0.000386701	0.000432653	0.985539878
N.Nitrosoguvacoline	23.71	5.66E-07	7.02E-07	0.000324815	0.103479511
4-(3-Hydroxybutyl)-2-methoxyphenyl hydrogen sulfate	15.31	2.38E-05	3.12E-05	0.002601555	0.278201571
X3..4.Methoxyphenyl.propyl.hydrogen.sulfate	14.57	3.46E-05	2.55E-05	0.471927573	0.001669411
Hexahydroxydiphenic acid	15.52	2.14E-05	0.000231309	0.000132199	0.998957001
1-Amino-1-deoxy-scyllo-inositol 4-phosphate	16.21	1.52E-05	0.001136197	2.63E-05	0.538383024
1-{[5-(2-Hydroxyethoxy)-4-oxopentanoyl]oxy}-2,5-pyrrolidinedione	20.81	1.86E-06	2.99E-05	1.57E-05	0.998823833
Mebutamate	18.57	4.99E-06	0.000156316	1.91E-05	0.86279313
N-(3,5-Dichlorophenyl)-N'-ethylthiourea	16.94	1.07E-05	0.195227498	6.41E-06	0.0037323
Maleic hydrazide	14.03	4.59E-05	0.001618216	9.50E-05	0.711421166
PEG n10	15.97	1.71E-05	0.067539046	9.78E-06	0.018632454
Triadimefon	21.71	1.27E-06	5.80E-06	4.90E-05	0.684905095
5,5'-Dihydroxy-4,4',8',8'-tetramethyl-4,5-dihydro-2'H,3H-spiro[furan-2,6'-[7]oxabicyclo[3.2.1]oct[3]en]-2'-one	32.32	2.61E-08	4.58E-08	0.897229466	7.54E-07
(1R,9S)-11-[(Methylsulfanyl)acetyl]-3-(2-thienyl)-7,11-diazatricyclo[7.3.1.02,7]trideca-2,4-dien-6-one	25.48	2.85E-07	1.06E-05	1.86E-06	0.923751101
5-(2,6-Dichlorobenzyl)-6-methyl-2-(2-pyridyl)pyrimidin-4-ol	24.24	4.60E-07	1.03E-05	4.08E-06	0.991315512

6-[(5-Amino-1-carboxypentyl)amino]-3,4,5-trihydroxytetrahydro-2H-pyran-2-carboxylic acid (non-preferred name)	21.17	1.60E-06	5.07E-05	7.97E-06	0.904041259
X7028	19.05	4.02E-06	2.18E-05	8.60E-05	0.829557536

Table S5: A total of 21 unique metabolite differences were observed between the dingo and Basenji. Pairwise differences between groups were obtained from Tukey's test.

Broad classification	Subclass
**Lipid**	
cis-5,8,11,14,17-Eicosapentaenoic acid	Fatty acid
Diallyl adipate	Fatty acid
Docosahexaenoic acid	Fatty acid
Nervonic acid	Fatty acid
14(Z)-Eicosenoic acid	Fatty acid
2-(2-Carboxyethyl)-4-methyl-5-pentyl-3-furoic acid	Fatty acid
LysoPC (22:1(13Z))	Lysophospholipid
LPC (22:6)	Phosphatidylcholine
PC (18:3/18:3)	Phosphatidylcholine
PC (16:0/22:5n3)	Phosphatidylcholine
**Protein**	
Hypoglycin A	Amino acid
Pro-Gln	Dipeptide
**Carbohydrate**	
Aminoimidazole ribotide	Carbohydrate
Glucose-1-phosphate	Carbohydrate
**Other**	
Uric acid	Purine derivative
N4-Acetylcytidine	Pyrimidine nucleoside
Dimetridazole	Drug
3-(4-Methoxyphenyl)propyl hydrogen sulfate	Phenylsulfates
5-Nonyl-2-oxotetrahydro-3-furancarboxylic acid	Gamma butyrolactones
6-Methoxyquinoline	Aromatic ether
5,5'-Dihydroxy-4,4',8',8'-tetramethyl-4,5-dihydro-2'H,3H-spiro[furan-2,6'-[7]oxabicyclo[3.2.1]oct[3]en]-2'-one	Unknown

Table S6: A total of 20 unique metabolite differences were observed between the dingo and German Shepherd Dog. Pairwise differences between groups were obtained from Tukey's test.

Broad classification	Subclass
**Protein**	
gamma-Glu-gln	Dipeptide
Gly-Pro(Glycylproline)	Dipeptide
L-Alanyl-L-proline	Dipeptide
(3S)-3_6-Diaminohexanoate	Amino acid derivative
2_6-Diaminoheptanedioic acid	Amino acid derivative
N5-(L-1-Carboxyethyl)-L-ornithine	Amino acid
N5-Ethyl-L-glutamine	Amino acid analogue
6-Acetamido-3-aminohexanoate	Beta-amino acids
L (+)-Citrulline	Amino acid
L-Homocitrulline	Amino acid derivative
N-Acetylornithine	Amino acid
**Carbohydrate**	
Benzoyl glucuronide (Benzoic acid)	Carbohydrate
**Other**	
N-(3,5-Dichlorophenyl)-N'-ethylthiourea	Synthetic
PEG n10	Synthetic polyether
Propamocarb	Drug
4-Amino-5-hydroxymethyl-2-methylpyrimidine	Pyrimidine
6-Hydroxypseudooxynicotine	Aryl alkyl ketones
D-1,2,3,4-Tetrahydroisoquinoline-3-carboxylic acid	Carboxylic acid
Methylimidazoleacetic acid	Imidazolyl carboxylic acids
N-((4-Amino-2-methyl-5-Pyrimidinyl) methyl) formamide	Amino pyrimidine
